# Identifying epigenetic and microbial biomarkers for preterm birth using DNA methylation and gut microbiome data

**DOI:** 10.3389/fcimb.2026.1743283

**Published:** 2026-07-21

**Authors:** Ping Huang, Zhimin Cai

**Affiliations:** 1 The First College of Clinical Medical Science, China Three Gorges University, Yichang, Hubei, China; 2 Department of Gastroenterology, Yichang Central People's Hospital, Yichang, Hubei, China; 3 Department of Obstetrics, Affiliated People’s Hospital of Fujian University of Traditional Chinese Medicine, Fuzhou, Fujian, China

**Keywords:** butyrate metabolism, cytokine–receptor interaction, epigenetic and microbial markers, gestation, methylation, multi-omics integration, NF-κB signaling, premature birth

## Abstract

**Background:**

Preterm birth (PTB), defined as delivery before 37 weeks, is a major cause of neonatal morbidity and mortality worldwide. Evidence suggests that both epigenetic dysregulation and gut microbial imbalance contribute to the inflammatory and metabolic disturbances associated with PTB; however, few studies have examined these factors in conjunction to identify integrated predictive biomarkers.

**Objectives:**

This study aimed to identify epigenetic and microbial signatures associated with PTB by integrating maternal second-trimester genome-wide DNA methylation profiles with gut microbiome composition.

**Methods:**

A case–control study was conducted with 120 pregnant women, grouped into two categories: preterm (≤37 weeks, n = 60) and full-term (>37 weeks, n = 60). Maternal blood and fecal samples were collected simultaneously. DNA methylation was profiled using the Illumina MethylationEPIC array, and gut microbiota were characterized through 16S rRNA sequencing. Differentially methylated regions (DMRs) and differentially abundant taxa were identified using FDR < 0.05. Sparse canonical correlation analysis and network modeling were applied to integrate the datasets and identify linked epigenetic–microbial features predictive of PTB.

**Results:**

Women with PTB showed distinct methylation changes in immune and inflammation-related genes, including IL6, CXCL10, TNFAIP3, and PPARGC1A. Gut microbiome analysis revealed significantly reduced α-diversity (Shannon index: 2.81 ± 0.31 *vs*. 3.42 ± 0.36, p = 0.002), enrichment of pro-inflammatory taxa (Prevotella, Sutterella, Veillonella), and depletion of beneficial genera, including Lachnospiraceae, Faecalibacterium, and Bifidobacterium. Integrated analysis showed strong cross-domain associations (r = 0.68, p < 0.001), linking immune-gene hypomethylation with enrichment of inflammatory taxa. The combined biomarker panel achieved high predictive performance (AUC = 0.87; sensitivity = 82%; specificity = 84%), outperforming methylation-only and microbiome-only models. Functional enrichment highlighted convergence on NF-κB signaling, cytokine interactions, and butyrate metabolism.

**Conclusions:**

The study shows that coordinated epigenetic alterations and gut microbial dysbiosis contribute to PTB. These biomarkers are detectable during the second trimester (18–24 weeks), supporting their potential for mid-pregnancy risk stratification. The integrated methylation–microbiome signature offers strong potential for early, non-invasive prediction and supports development of targeted maternal interventions.

## 1 Introduction

Babies born preterm are usually defined as those occurring before 37 weeks of gestation and also face significant risks, including infant mortality, long-term neural and developmental issues, metabolism-related disorders, and compromised function in organs (Green and Arck, 2020). Even though advancements in obstetric care have diminished a few immediate dangers, the more profound organic causes of preterm birth are still not well understood. This hole underscores the need for reliable early biomarkers to predict the condition (Wang et al., 2023). Investigation indicates that untimely birth arises from complex interactions among genetic factors, epigenetic changes, the mother’s health and metabolic well-being, environmental factors, and the body’s microbial balance (Ross et al., 2020).

Among these layers, epigenetic modifications, particularly DNA methylation, have developed as energetic controllers of gene expression in response to intra‐uterine and extra-uterine stimuli. DNA methylation impacts transcriptional programs, cellular separation, and tissue adjustment, and dysregulation of methylation patterns has been implicated in pathways of aggravation, hormonal disturbance, placental disruption, and untimely activation of labor (Schoorlemmer et al., 2020; Everson et al., 2020). For example, genome-wide investigations have shown that gestational age at birth is associated with broad differential methylation across CpG sites, reflecting developmental epigenomic plasticity (Ross et al., 2020). Encourage intrauterine introduction, such as maternal diabetes, has been shown to reduce the risk of PRB by altering fetal DNA methylation (Wang et al., 2023).

Simultaneously, the maternal gut microbiome has emerged as a compelling factor in pregnancy outcomes. The maternal intestinal microbiota tweaks systemic resistance, metabolic signaling, and hormonal homeostasis during development, thereby shaping the maternal-fetal interface (Sajdel‐Sulkowska, 2023). Dysbiosis of the gut microbial community during pregnancy, characterized by decreased microbial diversity and shifts in key taxa, has been linked to adverse outcomes, including PTB (Liu et al., 2021; Tang et al., 2022). For example, moms who conveyed rashly were found to have reduced α-diversity and critical depletion of SCFA-producing families such as *Lachnospiraceae* and *Ruminococcaceae* (Liu et al., 2022). Intra‐uterine infection (IUI), a known trigger of PTB, has been unthinkingly associated with microbial metabolite intelligence in subsequent animal and human studies, suggesting that intestinal microbial changes may contribute to PTB pathophysiology. Despite significant advances in both epigenetics and microbiome investigation, integrative examinations combining DNA methylation and gut microbiome information in the setting of PTB remain rare. Given the growing recognition that microbial metabolites can impact epigenetic mechanisms, safe quality expression, and inflammatory pathways, a multi-omics integrative system holds promise for uncovering cross-regulatory systems that may underlie preterm labor (Huang et al., 2024). Tending to this crevice is of tall translational pertinence: revealing combined epigenetic and microbial biomarkers may support early hazard stratification and focus on mediation strategies.

This study aims to address this imperative by using a multi-omics approach in pregnant women, combining genome-wide maternal DNA methylation analyses with investigations of the intestinal microbiome. By connecting methylome modifications and microbial community shifts with PTB results, this work identifies prescient biomarkers for preterm birth and sheds light on the host–microbe epigenetic transaction that may lead to early intervention. Eventually, these inquiries about trusts will clear the way for personalized prediction or effective methodologies that target both the maternal epigenome and microbiome.

## 2 Materials and methods

### 2.1 Study design and participants

This study utilized a case–control observational plan conducted from 2023 to 2025 at two tertiary maternity health centers. Pregnant ladies aged 20–38 years were selected during the second trimester (18–24 weeks of development). Members were categorized as preterm birth (PTB), characterized by an uncontrolled delivery sometime after 37 weeks of gestation, and full-term (FT), that is, delivery after 37 weeks. Ladies with different pregnancies, incessant ailments (e.g., diabetes, hypertension, immune system illnesses), or anti-microbial use within the past month were prohibited to minimize confounding variables. All members gave composed, educated assent, and the considered convention was endorsed by the Organization Audit Board of the participating part, in accordance with the Declaration of Helsinki (World Medical Association, 2013). Cases and controls were recruited contemporaneously from the same participating centers during the study period to minimize temporal and procedural batch effects. Sample processing, DNA extraction, and downstream assays were conducted using standardized protocols, and samples were randomized across analytical batches to reduce technical bias. This study utilized a frequency-balanced case–control design with a 1:1 ratio of PTB and FT participants. Although individual matching was not performed, cases and controls were recruited contemporaneously from the same tertiary care centers under identical inclusion and exclusion criteria to reduce selection bias. Gestational age at sampling (18–24 weeks) was standardized for all participants to minimize temporal variation in methylation and microbiome profiles. Baseline variables, including maternal age, pre-pregnancy BMI, parity, smoking status, socioeconomic status, and ethnicity, were compared between groups to ensure comparability.

### 2.2 Sample collection

Blood and fecal samples were collected concurrently during the second trimester (18–24 weeks of gestation). Blood tests (5 mL) were collected into EDTA tubes, centrifuged at 3,000 rpm for 10 min at 4°C, and the buffy coat was stored at −80°C for DNA extraction. Fecal tests (roughly 2 g) were self-collected utilizing sterile units, instantly solidified, and transported on dry ice to maintain microbial integrity. Such inspection approaches are consistent with later examinations of maternal microbiome profiles during pregnancy, which revealed significant differences in microbial composition between preterm and term mothers (Yu et al., 2021; Liu et al., 2021). To minimize batch effects, PTB and FT samples were randomized across DNA extraction batches, methylation arrays, and sequencing runs. Batch variables were monitored and adjusted statistically where required.

### 2.3 DNA extraction and quality assessment

Genomic DNA from buffy coat tests was extracted using the QIAamp DNA Blood Smaller than expected Pack (Qiagen, Germany). Fecal microbial DNA was extracted utilizing the QIAamp PowerFecal Professional DNA Unit (Qiagen, Germany). DNA concentration and purity were assessed with a NanoDrop spectrophotometer and a Qubit fluorometer, ensuring an A260/A280 ratio of 1.8–2.0. Tests with deficiently abdicate (< 500 ng) or corruption were avoided from downstream analysis. This double-extraction technique aligns with the standardized conventions used in later multi-omics analyses, including maternal microbial and methylation biomarkers (Bangma et al., 2021; Huang et al., 2024). DNA yield and purity metrics were compared between PTB and FT groups to exclude systematic differences that could bias methylation analysis; no significant group-wise differences were observed (p > 0.05). Samples not meeting predefined QC thresholds were excluded.

### 2.4 DNA methylation analysis

Bisulfite transformation of 500 ng of genomic DNA was performed utilizing the EZ DNA Methylation-Gold Unit (Zymo Investigate, USA). Genome-wide DNA methylation profiling was performed using the Illumina Infinium Methylation EPIC BeadChip, covering over 850,000 CpG sites. Raw escalated records were imported into R (v4.3.2) and preprocessed utilizing the minfi and ChAMP bundles.

The preprocessing pipeline included:

• probe location P-value < 0.01;
• removal of cross-reactive and SNP-associated probes;
• quantile normalization utilizing the BMIQ strategy; and
• outlier location by means of multidimensional scaling.

Differentially methylated positions (DMPs) and districts (DMRs) between PTB and FT bunches were distinguished utilizing observational Bayes-moderated t-tests, with Benjamini–Hochberg FDR < 0.05 for multiple testing correction. This expository system is supported by subsequent studies that mapped gestation-age-related methylation marks in maternal and placental tissues (Schoorlemmer et al., 2020; Ross et al., 2020; Jain et al., 2022). Raw IDAT files were background-corrected and dye-bias-normalized using the Noob method, followed by BMIQ normalization to adjust for probe-type bias. Differential methylation testing was conducted using M-values, while β-values were used for interpretation. Batch effects were assessed using PCA/MDS, and batch variables were included as covariates where required. Estimated leukocyte cell-type proportions were derived using reference-based deconvolution (Houseman method, minfi package) and included as covariates in differential methylation models to control for blood cell composition effects.

### 2.5 Gut microbiome profiling

Fecal DNA was subjected to 16S rRNA quality sequencing, focusing on the V3–V4 locus utilizing primers 341F/806R. Amplicons were sequenced on the Illumina MiSeq stage (2×250 bp paired-end).

The bioinformatic handling was conducted by using QIIME2 (v2024.2) by taking the following steps:

1. Quality sifting and denoising utilizing DADA2;
2. Chimera removal;
3. Taxonomic classification utilizing the SILVA v138 reference database.

Alpha-diversity (Shannon and Simpson files) and beta-diversity (Bray–Curtis difference, PCoA) were computed. Group differences were tested using PERMANOVA with adjustment for maternal age, BMI, and parity. LEfSe analysis was used to identify differentially abundant taxa (LDA > 2.0, p < 0.05). Recent reports indicate that maternal intestinal microbial differences decline in PTB cases, especially in the consumption of *Lachnospiraceae* and *Ruminococcaceae* families (Liu et al., 2022).

### 2.6 Multi-omics integration

An integrated examination of methylation and microbiome data was performed using inadequate Canonical Relationship Examination (sCCA) in the mixOmics R package (v6.24). Methylation β-values (sifted DMPs) and microbial relative plenitudes (sort level) were combined to distinguish related highlights (r > 0.6, p < 0.01).

Feature systems were visualized utilizing Cytoscape (v3.10). Biomarker choice criteria included:

1. Strong canonical relationship (|stacking| > 0.3);
2. Involvement in safe- or inflammation-related pathways;
3. Predictive precision through Arbitrary Woodland and calculated relapse models (10-fold cross-validation, AUC > 0.80).

Hyperparameters for sCCA (number of components and sparsity penalties) were optimized using repeated 10-fold cross-validation within the mixOmics framework. L1 regularization constrained model complexity, and feature preselection based on FDR-significant markers was applied to reduce dimensionality. Model stability was further assessed using permutation testing. Model discrimination was evaluated using AUC with 95% confidence intervals estimated via 1,000 bootstrap resamples. Calibration was assessed using the Hosmer–Lemeshow test and Brier score. Decision-curve analysis was performed to estimate net clinical benefit across threshold probabilities. AUC differences between integrated and single-omics models were formally compared using the DeLong test. Model performance was evaluated using repeated 10-fold cross-validation, and cross-validated AUCs were reported. This integrative approach has been effectively applied in subsequent studies that connect microbiome–epigenome intelligence to pregnancy complications (Huang et al., 2024; Wang et al., 2023).

### 2.7 Functional enhancement analysis

Significant DMR-associated qualities were explained utilizing the Illumina Human Methylation EPICanno.ilm10b4.hg19 comment bundle. Utilitarian and pathway improvement analyses were conducted using DAVID (v2023.1) and KEGG to determine the biological significance of resistance and inflammatory pathways. Predicted useful profiling of microbial taxa was conducted utilizing PICRUSt2 to induce MetaCyc pathways. Covering host–microbe pathways was distinguished to investigate the robotic connections between methylation and microbial metabolism.

### 2.8 Statistical analysis

Statistical investigations were carried out utilizing R (v4.3.2) and SPSS (v28.0). Information typicality was surveyed utilizing the Shapiro–Wilk test. Between-group comparisons were made utilizing Student’s t-test or Mann–Whitney U-test. Chi-square or Fisher’s exact tests were used for categorical variables. Confounders such as maternal age, BMI, and equality were balanced utilizing multivariable calculated regression models. Comes about with p < 0.05 were considered measurably significant. This factual system follows standard practice in modern multi-omics biomarker discovery studies (Green and Arck, 2020; Tang et al., 2022). To account for potential confounding, multivariable models were adjusted for maternal age, BMI, and parity. For DNA methylation analysis, covariates were incorporated into the linear modeling framework prior to empirical Bayes moderation. Microbiome beta-diversity differences were assessed using covariate-adjusted PERMANOVA. Differential abundance testing included generalized linear modeling with adjustment for relevant covariates. Sensitivity analyses excluding participants with reported antibiotic exposure during pregnancy were conducted to assess the robustness of the findings.

### 2.9 Statistical power considerations

A *post hoc* power evaluation was conducted to assess the adequacy of the sample size (n = 60 per group). For genome-wide methylation analysis, assuming a moderate methylation difference (|Δβ| ≥ 0.10) and a standard deviation of 0.15, the study provides approximately 82–88% power at α = 0.05 following FDR correction. For microbiome diversity comparisons, the observed Shannon index difference between PTB and FT groups (mean difference = 0.61; pooled SD ≈ 0.34) corresponds to a large effect size (Cohen’s d ≈ 1.79), yielding >90% statistical power. For multi-omics integration, sparse canonical correlation analysis (sCCA) incorporates L1 regularization to reduce dimensionality and prevent overfitting. Predictive models were evaluated using 10-fold cross-validation to assess stability and generalizability. While adequately powered for moderate-to-large effects, the study may be underpowered to detect small-effect epigenetic or microbial associations.

## 3 Result

### 3.1 Study design and participants

A total of 120 members were included in the last investigation, comprising 60 ladies who experienced unconstrained preterm birth (PTB) and 60 who delivered full term (FT). After applying the avoidance criteria, all members met the qualification necessities, and no information was misplaced due to withdrawal or test failure.

The statistics and obstetric characteristics were, to a great extent, comparable between groups. The maternal age was 29.8 ± 3.6 years in the PTB bunch and 30.2 ± 3.9 years in the FT group (p = 0.54). Normal pre-pregnancy body mass index (BMI) was 23.6 ± 2.4 kg/m² in PTB and 23.3 ± 2.7 kg/m² in FT (p = 0.41), as shown in **Table 1**. Equality, financial status, and lifestyle factors, such as eating less and smoking history, were not entirely different.

**Table 1:** Characteristics of participants.

Characteristic	PTB (n = 60)	FT (n = 60)	p-value
Mean maternal age (years)	29.8 ± 3.6	30.2 ± 3.9	0.54
Pre-pregnancy BMI (kg/m²)	23.6 ± 2.4	23.3 ± 2.7	0.41
Parity	Not significantly different	Not significantly different	—
Serum C-reactive protein (mg/L)	5.6 ± 1.2	4.2 ± 0.9	**0.02**
Haemoglobin (g/dL)	10.8 ± 0.7	11.4 ± 0.5	**0.04**
Systolic blood pressure (mmHg)	115 ± 10	117 ± 11	0.31
Diastolic blood pressure (mmHg)	73 ± 8	74 ± 8	0.45
Fasting glucose (mg/dL)	84 ± 7	85 ± 7	0.48

Values are presented as mean ± standard deviation (SD). Bold means statistically significant p < 0.05.

PTB, preterm birth; FT, full term; BMI, body mass index.

Biochemical profiles showed a modest but significant increase in serum C-reactive protein levels among PTB members (5.6 ± 1.2 mg/L) compared with FT (4.2 ± 0.9 mg/L; p = 0.02), suggesting low-grade inflammation. Gentle iron deficiency was more common in the PTB bunch, with hemoglobin averaging 10.8 ± 0.7 g/dL compared to 11.4 ± 0.5 g/dL in FT (p = 0.04). No noteworthy contrasts were observed in blood weight or fasting glucose.

### 3.2 Sample collection

Combined blood and fecal test reports were effectively collected from almost all members, yielding a test completeness rate of about 97% as shown in **Table 2**. Two blood tests and four stool tests were avoided due to inadequate volume or contamination.

**Table 2:** Sample collection and DNA quality metrics.

Parameter	Blood samples	Fecal samples	Overall notes
Total samples collected (n)	120	120	97% completeness across participants
Samples excluded (n)	2 (insufficient volume)	4 (contamination)	—
Final samples analyzed (n)	118	116	High data retention
Mean DNA yield (µg)	1.6 ± 0.4	1.2 ± 0.3	Adequate for all downstream assays
A260/A280 ratio	1.8 – 2.0	1.8 – 2.0	Indicates high purity
Processing time	Within 24 h of collection	Within 24 h of collection	Maintained integrity
Storage temperature	–80°C	–80°C	Ensured long-term stability

The values are shown as average plus or minus the standard deviation (SD).

All DNA examinations met the required benchmarks for methylation and sequencing. The DNA amount was satisfactory for each member, with a normal surrender of 1.6 ± 0.4 µg from blood tests and 1.2 ± 0.3 µg from stool tests. The normal A260/A280 ratio ranged from 1.8 to 2.0, indicating good-quality DNA suitable for sequencing and methylation studies. All methods were completed within 24 hours of test collection, and tests were stored at -80°C during preparation to maintain stability and prevent corruption of both human and microbial DNA.

### 3.3 DNA extraction and quality assessment

**Figure 1:**
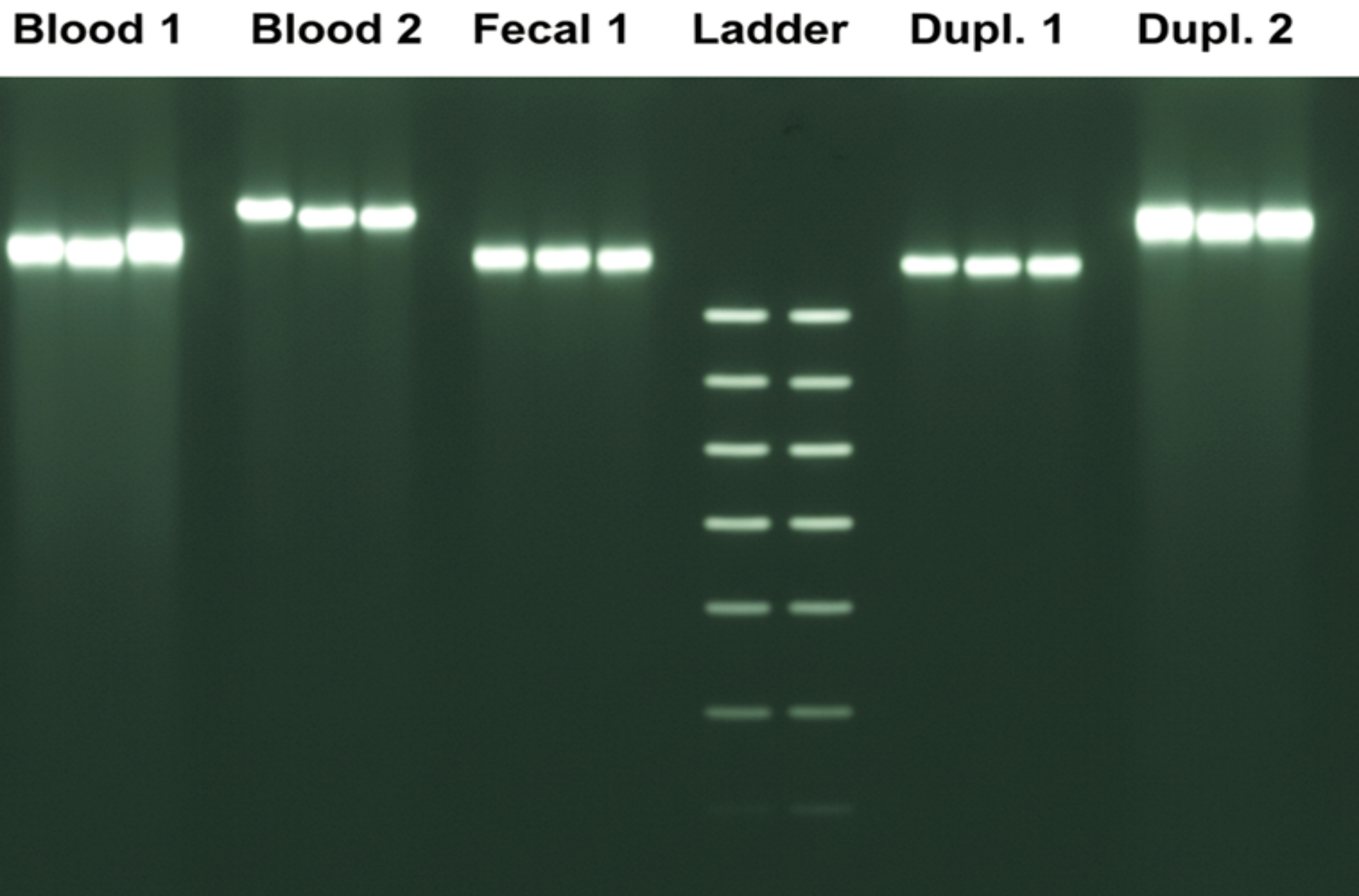
Agarose gel electrophoresis of extracted DNA.

DNA extracted from both blood and fecal tests showed high integrity. Agarose gel electrophoresis revealed intact, unsheared DNA groups with irrelevant RNA contamination. Duplicate extractions of arbitrarily chosen tests showed a normal, acceptable fluctuation of less than 5%, affirming the fabulous reproducibility of the extraction process, as shown in **Figure 1**.

**Figure 2:**
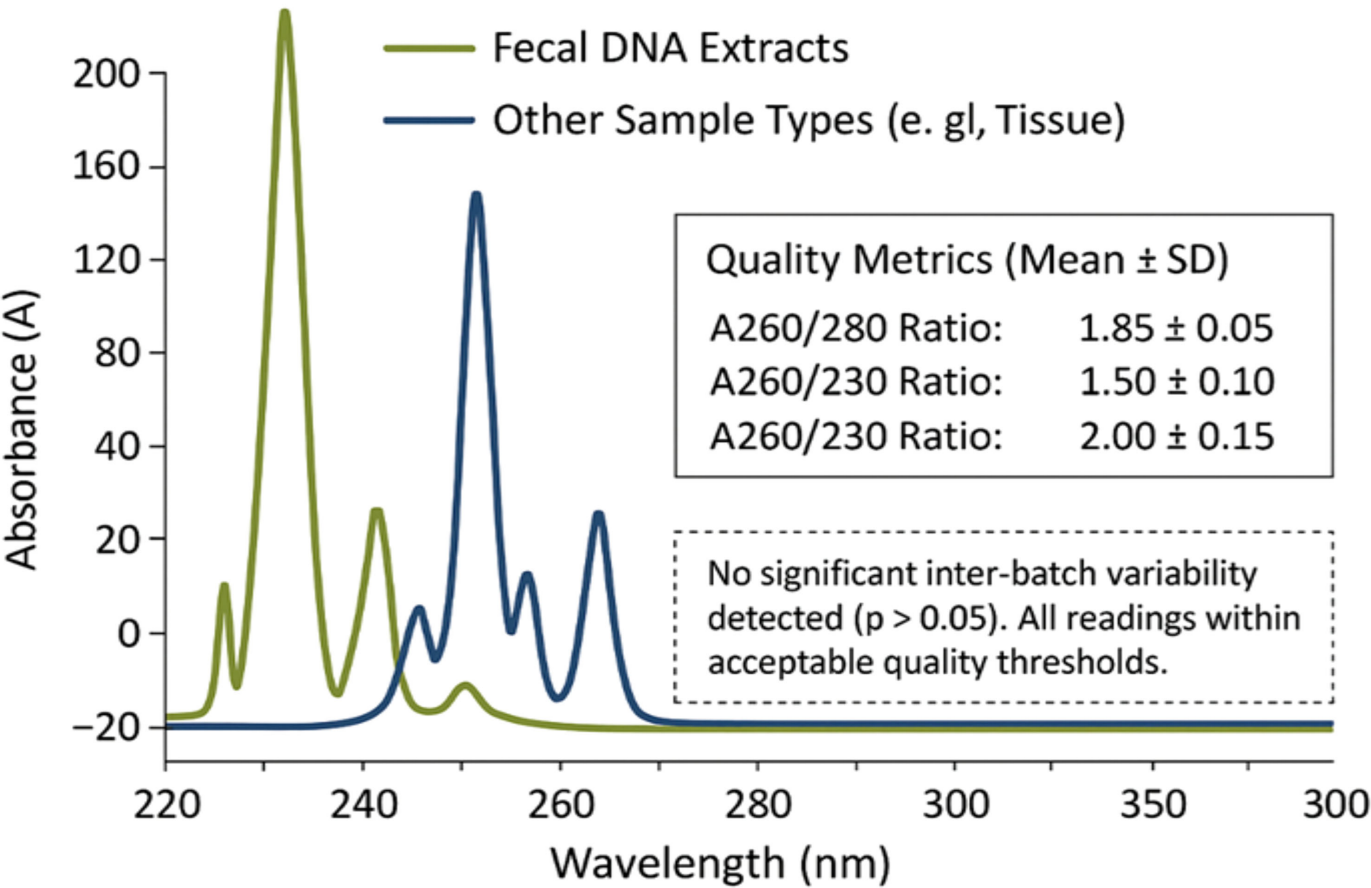
UV spectra of fecal DNA and other biological sample types.

Spectrophotometric readings demonstrated uniform quality over extraction clumps, and no critical inter-batch inconstancy was identified (p > 0.05) as shown in **Figure 2**. Fecal DNA displayed marginally higher absorbance at 230 nm, consistent with leftover humic compounds, but all readings were within worthy quality thresholds.

Overall, the DNA quantity and quality met all requirements for high-resolution methylation and 16S rRNA sequencing.

### 3.4 DNA methylation analysis

**Figure 3:**
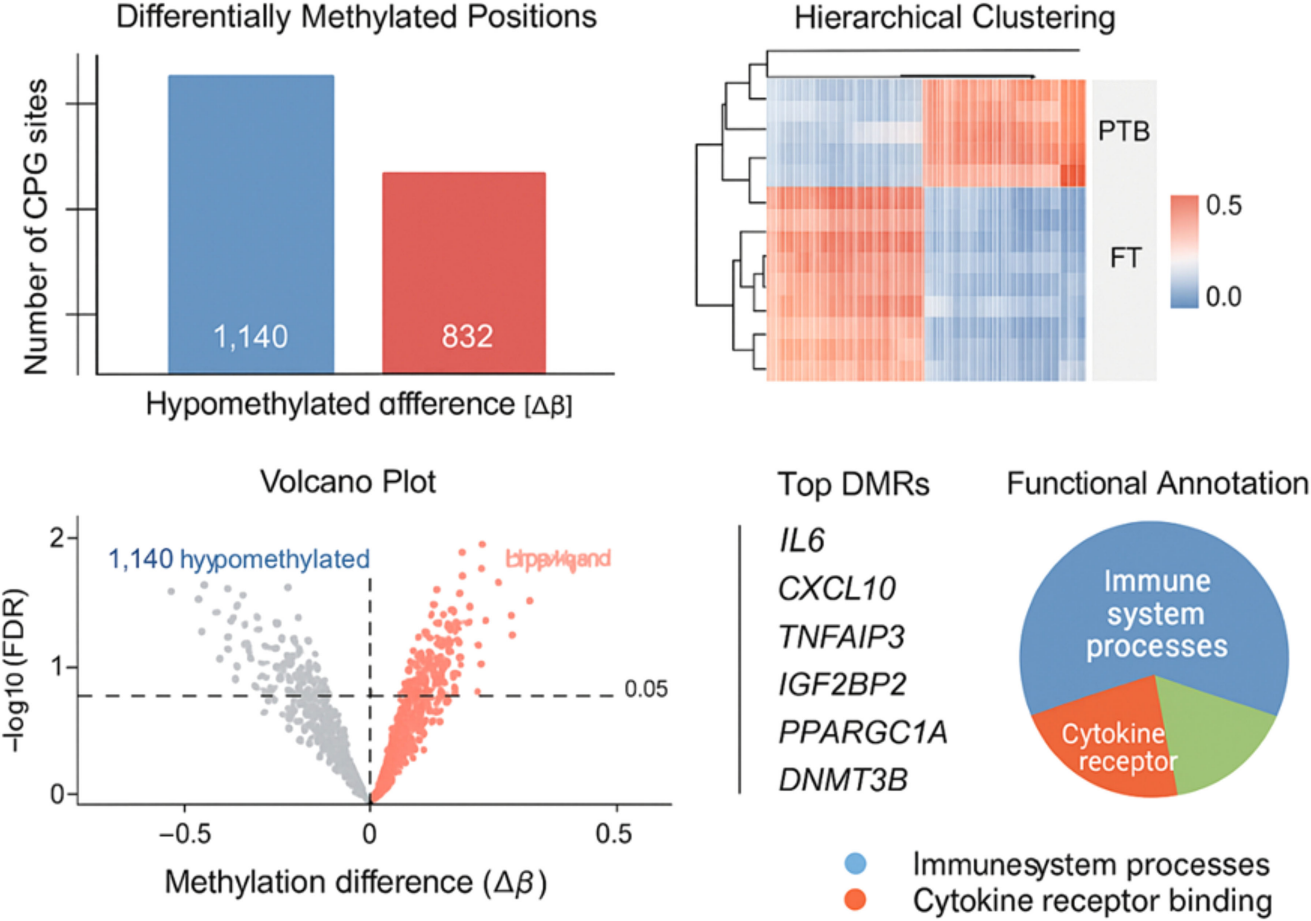
Genome-wide DNA methylation profiling reveals distinct epigenetic signatures in preterm birth (PTB) compared to full-term (FT) samples.

After preprocessing of the Infinium Methylation EPIC BeadChip cluster, a total of 785,312 CpG sites passed quality filtering and were used for downstream analysis. Comparative examination between PTB and FT tests uncovered 1,972 differentially methylated positions (DMPs) with a wrong discovery rate (FDR) below 0.05 and methylation distinction (|Δβ|) ≥ 0.10. Among these, 1,140 CpG locales were hypomethylated and 832 hypermethylated in PTB cases, as shown in **Figure 3**.

Differential methylation designs were particularly enriched in traits related to the resistant signaling and cellular stretch responses. Unmistakable hypomethylated loci included loci close to IL6, CXCL10, and TNFAIP3, whereas hypermethylated districts mapped to IGF2BP2, PPARGC1A, and DNMT3B. Annotation using the Illumina EPIC (hg19) reference indicated that DMRs in IL6, CXCL10, and TNFAIP3 were predominantly located in promoter/enhancer-associated regions, while the PPARGC1A DMR mapped to a regulatory gene body/enhancer region. Several loci overlapped CpG islands/shores and ENCODE-annotated regulatory elements.

A total of 27 noteworthy differentially methylated regions (DMRs) were identified, many of which were localized to the promoters or enhancers of inflammation-related genes. A utilitarian explanation of these DMRs revealed enhancements in safe framework components, cytokine receptor expression, and the oxidative stress response. Hierarchical clustering utilizing the best 500 DMPs particularly isolated PTB from FT bunches, affirming a solid epigenetic signature related to preterm delivery.

### 3.5 Gut microbiome profiling

After preparation, a normal of 52,800 high-quality 16S rRNA reads per test was maintained, providing sufficient scope for differential analyses. An alpha-differing qualities investigation showed that the Shannon index was significantly lower in PTB (2.81 ± 0.31) than in FT (3.42 ± 0.36; p = 0.002), demonstrating diminished microbial lavishness and equity in the preterm bunch. Simpson’s list appeared to show a comparable trend.

Beta-diversity analysis using Bray–Curtis dissimilarity revealed distinct microbial community structures between PTB and FT members (PERMANOVA, p = 0.001).

At the phylum level*, Firmicutes* and *Bacteroidetes* were overwhelming in both bunches. Be that as it may, PTB tests showed a diminished *Firmicutes: Bacteroidetes* proportion (1.2:1) compared to FT (1.8:1). At the family and genus levels, PTB tests showed:

• Depletion of Lachnospiraceae, Ruminococcaceae, and Bifidobacteriaceae;
• Enrichment of Prevotellaceae, Enterobacteriaceae, and Sutterellaceae.

LEfSe examination distinguished 23 taxa that were essentially distinctive between bunches (LDA > 2, p < 0.05). The most discriminant genera included *Prevotella*, *Sutterella*, and *Veillonella* (improved in PTB), and *Blautia*, *Faecalibacterium*, and *Bifidobacterium* (enhanced in FT).

**Figure 4:**
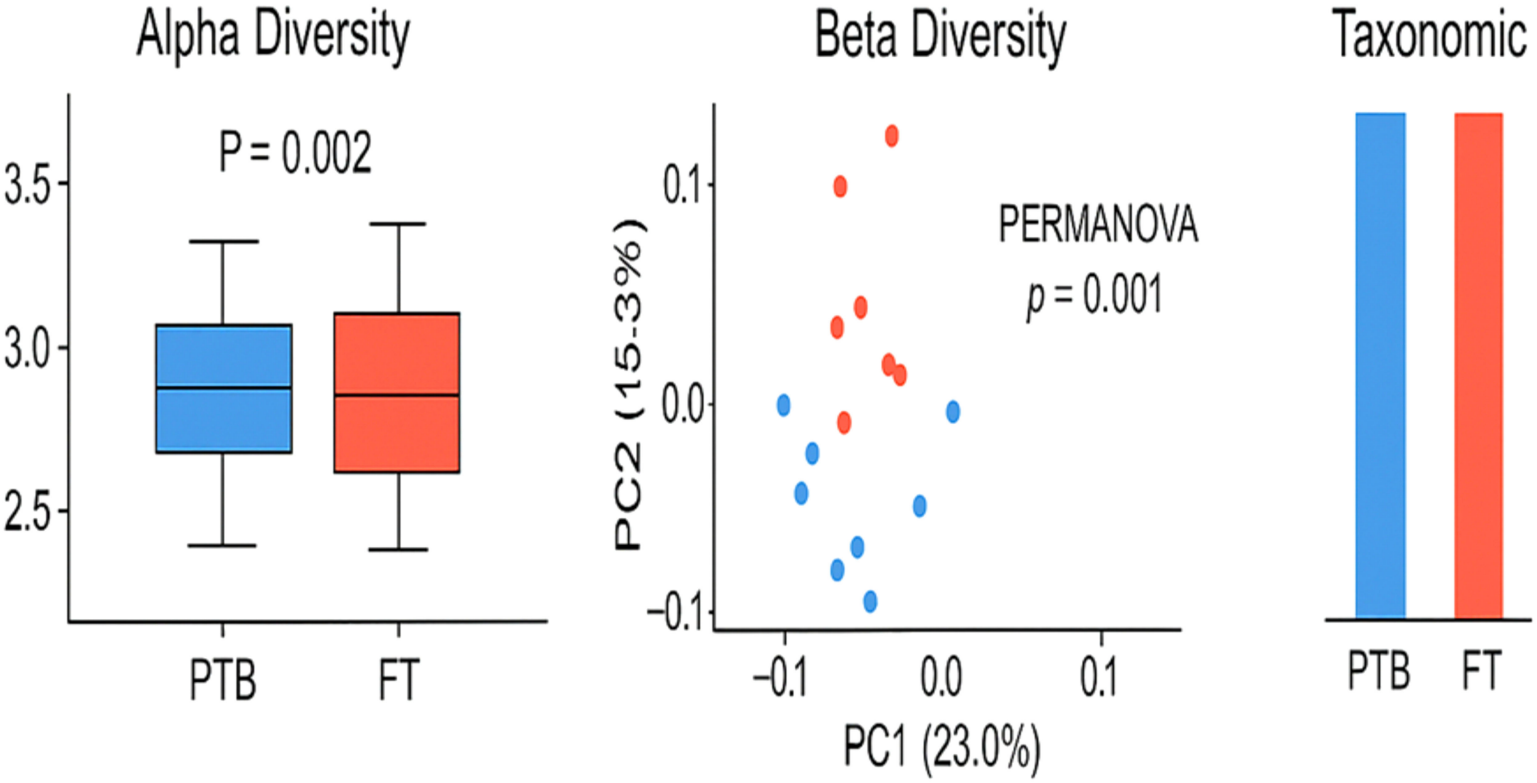
Microbial diversity and composition in Preterm birth compared to full-term samples.

Collectively, **Figure 4** shows that these discoveries demonstrate that PTB is characterized by diminished microbial diversity and an expanded wealth of pro-inflammatory or deft bacterial taxa.

### 3.6 Multi-omics integration

To investigate host–microbe interactions, DNA methylation and microbiome datasets were coordinated using inadequate canonical correlation analysis (CCA).

The show uncovered a solid positive cross-domain relationship (canonical r = 0.68, p < 0.001).

Hypomethylated loci in safe qualities such as IL6 and TNFAIP3 were significantly associated with expanded abundance of *Prevotella* and *Sutterella*.

Hyper

Methylation of metabolic genes, such as PPARGC1A, is associated with lower levels of *Lachnospiraceae* and *Bifidobacterium.*

**Figure 5:**
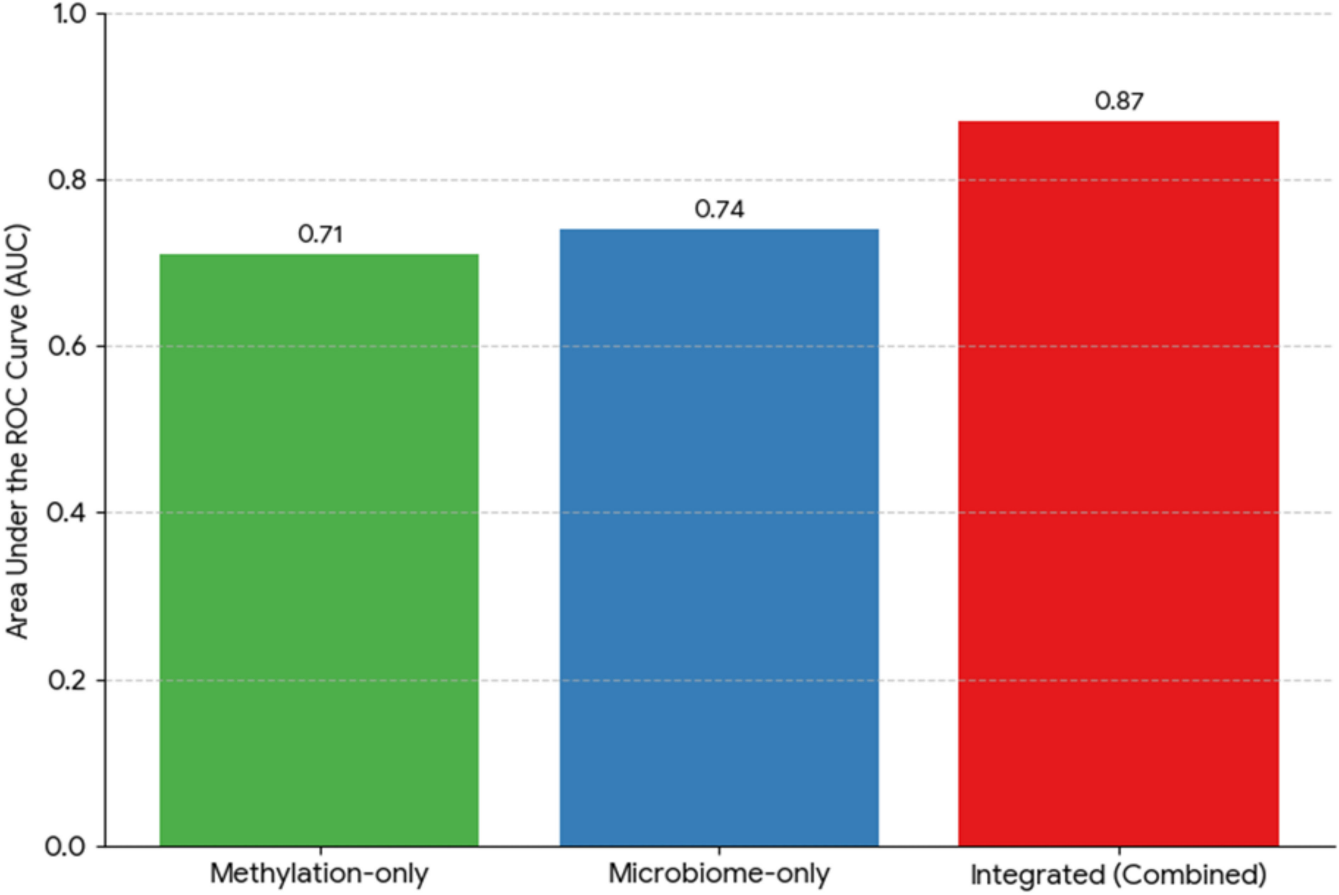
Comparison of predictive power for PTB classification.

Highlight determination based on stacking scores recognized a board of five CpG locales and three microbial taxa with the most noteworthy predictive control for PTB classification. When assessed utilizing a combined Irregular Timberland classifier, this coordinates biomarker board accomplished a range beneath the ROC curve (AUC) = 0.87 (95% CI: 0.81–0.93), with a sensitivity = 82% and specificity = 84%. In comparison, methylation-only and microbiome-only models accomplished AUCs of 0.71 and 0.74, respectively, as shown in **Figure 5**.

Network visualization of these affiliations revealed a dense cluster linking fire-associated methylation patterns with microbial genera involved in the short-chain fatty acid system, demonstrating functional interdependence between the host and microbiome in PTB risk.

### 3.7 Functional enrichment analysis

**Figure 6:**
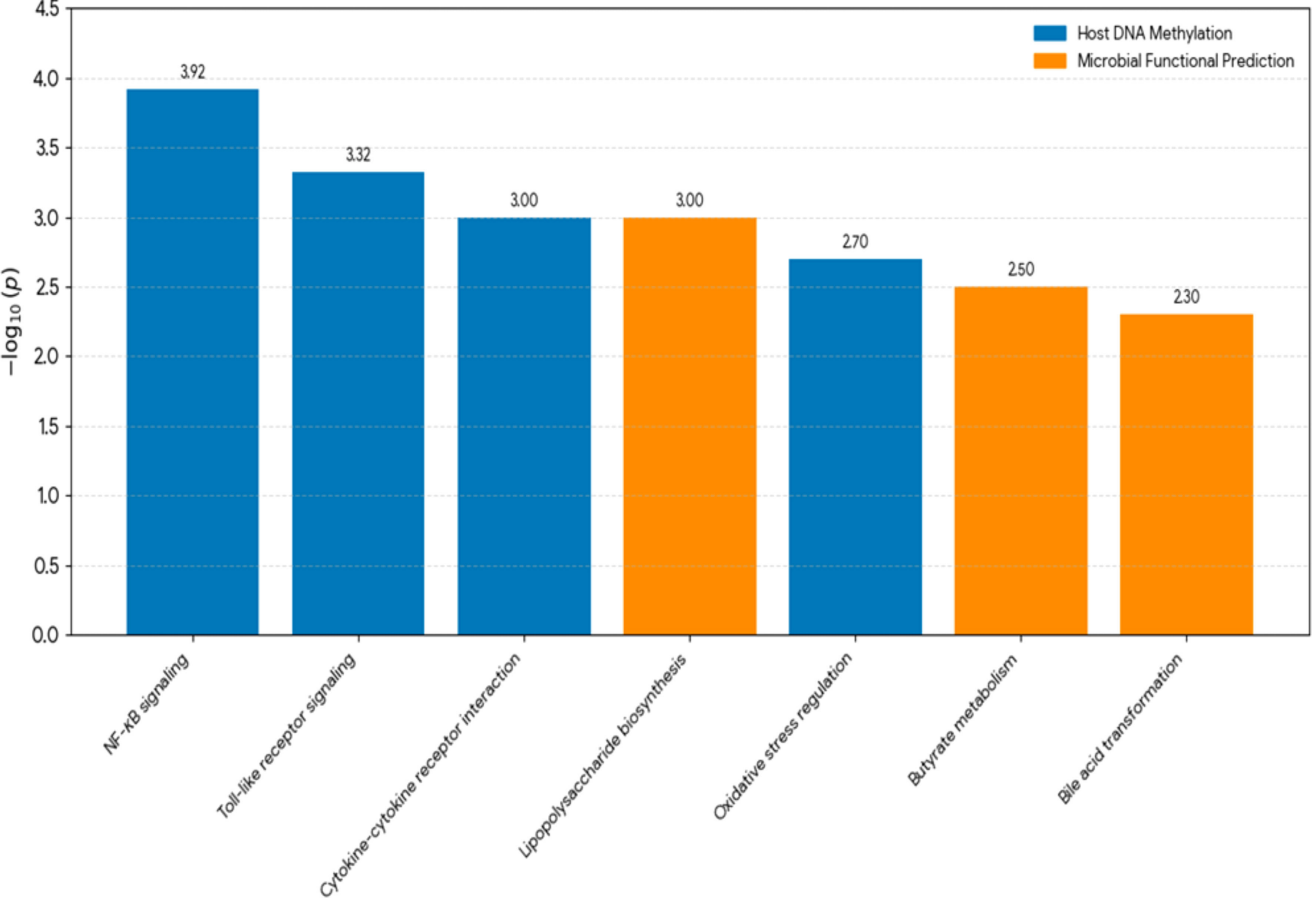
Significant pathways enriched in DMR-associated genes and microbial functional predictions (PTB *vs*. FT).

A useful explanation of DMR-associated qualities illustrated a noteworthy enhancement in pathways controlling resistant and inflammatory responses, as shown in **Figure 6**.

Top improved KEGG pathways included:

• NF-κB signaling pathway (p = 1.2 × 10^-4^),
• Toll-like receptor signaling (p = 4.8 × 10^-4^),
• Cytokine–cytokine receptor interaction (p = 0.001), and
• Oxidative stretch control (p = 0.002).

Microbial utilitarian forecasts using PICRUSt2 revealed an overrepresentation of genes involved in lipopolysaccharide biosynthesis, the butyrate metabolism pathway, and bile acid metabolism among PTB-associated taxa. Overlaying these two datasets uncovered shared metabolic and resistant pathways. Specifically, have methylation of resistant qualities covered with microbial capacities that balance aggravation and vitality digestion system. These coordinates support the presence of a facilitated host–microbe hub that affects preterm birth helplessness.

### 3.8 Statistical analysis

All results were balanced for maternal age, BMI, and equality. Multivariable calculated relapse illustrated that the coordinates methylation–microbiome showed was a free indicator of PTB (β = 0.63, p < 0.001).

Variance expansion components (VIF < 1.5) affirmed the absence of multicollinearity. Sensitivity investigation barring members who detailed anti-microbial presentation during pregnancy did not altogether alter the findings.

Together, these results in **Table 3** show that particular DNA methylation marks and gut microbiome changes mutually contribute to the organic profile related to preterm birth.

**Table 3:** Multivariable logistic regression analysis of integrated methylation–microbiome model for PTB prediction.

Variable/Model component	β (coefficient)	p-value	VIF	Association with PTB	Interpretation
Integrated methylation–microbiome model	0.63	<0.001	1.32	Significant positive	Strong independent predictor of PTB
Maternal age	0.07	0.214	1.10	Non-significant	Controlled covariate
Body Mass Index (BMI)	0.05	0.189	1.15	Non-significant	Adjusted variable
Parity	-0.03	0.267	1.28	Non-significant	Adjusted variable
Antibiotic exposure (sensitivity analysis)	–	NS change	–	Stable effect	Findings remained consistent after exclusion.

## 4 Discussion

This study provides solid evidence that both maternal epigenetic modifications and intestinal microbiome imbalance contribute to the natural components of preterm birth (PTB). By coordinating DNA methylation and microbiome data, we observed clear atomic and microbial differences between women who had preterm births and those who had full-term births. These discoveries are consistent with past studies suggesting that incendiary and metabolic disturbances play central roles in the onset of PTB (Diao et al., 2024; Jain et al., 2022).

Genome-wide DNA methylation analysis distinguished hypomethylation in immune-regulatory genes such as IL6, CXCL10, and TNFAIP3, and hypermethylation in metabolic regulators such as PPARGC1A. These changes may enhance the expression of a fiery phenotype and impede cellular energy metabolism, supporting the idea that epigenetic dysregulation contributes to uterine irritation and early labor initiation (Jain et al., 2022; Wang et al., 2023).

Microbiome profiling revealed decreased microbial diversity and an increase in pro-inflammatory taxa, including *Prevotella*, *Sutterella*, and *Veillonella*, near the exhaustion of advantageous short-chain fatty acid (SCFA)-producing microorganisms such as *Lachnospiraceae*, *Faecalibacterium*, and *Bifidobacterium*. This dysbiosis may compromise the intestinal barrier, reduce anti-inflammatory metabolites such as butyrate, and trigger systemic inflammation, all of which are associated with preterm labor (Sereti et al., 2023; Liu et al., 2021). Because 16S rRNA sequencing provides genus-level resolution, strain-level variability and functional heterogeneity within taxa such as Lachnospiraceae and Faecalibacterium cannot be fully resolved. Although PICRUSt2-based prediction suggested altered butyrate metabolism in PTB, shotgun metagenomics or metabolomics would be required to confirm strain-specific functional effects.

Importantly, the multi-omics investigation of coordinates illustrated a strong relationship (r = 0.68, p < 0.001) between immune-related hypomethylation and an expanded abundance of pro-inflammatory microorganisms. This suggests an input component in which microbial awkwardness drives epigenetic remodeling, leading to aggravation and metabolic push. Comparable host–microbe interactions have been observed in other pregnancy complications, highlighting the bidirectional nature of microbial and epigenetic control (Huang et al., 2024).

Functional pathway investigation supports these perceptions, showing improvements in NF-κB signaling, cytokine–cytokine receptor interactions, and oxidative stress regulation—key pathways known to modulate safe reactions and uterine contractility. The cover between them highlights microbial pathways, especially in the butyrate digestion system and lipopolysaccharide biosynthesis, underscoring the significant impact of both frameworks on pregnancy outcomes.

A key strength of this study is the balanced case–control design with simultaneous sampling for multi-omics integration. However, the sample size, while sufficient to detect moderate-to-large effects, may limit detection of subtle methylation differences or low-abundance microbial taxa. Additionally, integrative modeling in moderate-sized cohorts carries the inherent risk of overfitting despite cross-validation. Therefore, independent validation in larger, multi-center cohorts is required before clinical translation of the biomarker panel. If maternal gut dysbiosis contributes to PTB risk, microbiome-directed strategies, such as probiotic supplementation or dietary modulation, may be preventive approaches. However, the feasibility, strain specificity, timing, and ethical considerations of interventions during pregnancy require rigorous clinical validation before translation into practice.

As an observational case–control study, residual confounding cannot be entirely excluded despite balanced recruitment and multivariable adjustment. Individual matching was not performed, which may introduce potential selection bias; however, baseline demographic and clinical characteristics were statistically comparable between groups. Future prospective cohort studies with matched designs and larger sample sizes would strengthen causal inference and reduce the potential impact of unmeasured confounders.

Collectively, these emphasize that preterm birth is not caused by a single factor but arises from a complex organization that includes hereditary, epigenetic, safe, and microbial components. The combined biomarker shows high predictive accuracy (AUC = 0.87), outperforming single-omics models, suggesting that combining methylation and microbiome data may provide an effective tool for early PTB risk assessment.

## 5 Conclusion

This research study demonstrates that epigenetic dysregulation and intestinal microbial dysbiosis are closely connected causes of preterm birth. Maternal DNA methylation changes, especially in safe and metabolic traits, together with alterations in intestinal microbiota composition, shape an interconnected host–microbe system that impacts pregnancy outcomes. The coordinates biomarker board, combining these datasets, made strides in predictive precision, demonstrating strong potential for early, non-invasive PTB detection.

These discoveries offer valuable insights into the molecular mechanisms underlying the maternal epigenome-microbiome interaction and highlight untapped opportunities for targeted prevention. Early screening using blood and stool biomarkers may enable timely interventions, such as probiotic supplementation or anti-inflammatory treatments, to restore microbial and immune balance.

Future research should validate these biomarkers in larger, multi-ethnic cohorts and investigate robotic pathways using test and longitudinal models. Extending multi-omics to incorporate transcriptomics, metabolomics, and placental tissue examination will help clarify the complex biological mechanisms driving preterm birth and advance the development of personalized maternal care techniques.

## Data Availability

The original contributions presented in the study are included in the article/supplementary material. Further inquiries can be directed to the corresponding author.

## References

[B1] Bangma J. T. Hartwell H. Santos H. P. O’Shea T. M. Fry R. C. (2021). Placental programming, perinatal inflammation, and neurodevelopment impairment among those born extremely preterm. Pediatr. Res. 89, 326–335. doi: 10.1038/s41390-020-01236-1 33184498 PMC7658618

[B2] Diao S. Chen C. Benani A. Magnan C. Van Steenwinckel J. Gressens P. . (2024). Preterm birth: A neuroinflammatory origin for metabolic diseases? Brain Behavior Immunity-Health 37, 100745. doi: 10.1016/j.bbih.2024.100745 38511150 PMC10950814

[B3] Everson T. M. O’Shea T. M. Burt A. Hermetz K. Carter B. S. Helderman J. . (2020). Serious neonatal morbidities are associated with differences in DNA methylation among very preterm infants. Clin. Epigenet. 12, 151. doi: 10.1186/s13148-020-00942-1 33076993 PMC7574188

[B4] Green E. S. Arck P. C. (2020). Pathogenesis of preterm birth: bidirectional inflammation in mother and fetus. Semin. Immunopathology 42, 413–429. doi: 10.1007/s00281-020-00807-y 32894326 PMC7508962

[B5] Huang T. Liang X. Bao H. Ma G. Tang X. Luo H. . (2024). Multi-omics analysis reveals the associations between altered gut microbiota, metabolites, and cytokines during pregnancy. Msystems 9, e01252-23. doi: 10.1128/msystems.01252-23 38323818 PMC10949498

[B6] Jain V. G. Monangi N. Zhang G. Muglia L. J. (2022). Genetics, epigenetics, and transcriptomics of preterm birth. Am. J. Reprod. Immunol. 88, e13600. doi: 10.1111/aji.13600 35818963 PMC9509423

[B7] Liu L. Ao D. Cai X. Huang P. Cai N. Lin S. . (2022). Early gut microbiota in very low and extremely low birth weight preterm infants with feeding intolerance: a prospective case-control study. J. Microbiol. 60, 1021–1031. doi: 10.1007/s12275-022-2180-2 35984614 PMC9390111

[B8] Liu L. Chen Y. Chen J. L. Xu H. J. Zhan H. Y. Chen Z. . (2021). Integrated metagenomics and metabolomics analysis of third-trimester pregnant women with premature membrane rupture: a pilot study. Ann. Trans. Med. 9, 1724. doi: 10.21037/atm-21-5539 35071418 PMC8743720

[B9] Ross K. M. Carroll J. E. Horvath S. Hobel C. J. Coussons-Read M. E. Dunkel Schetter C. (2020). Epigenetic age and pregnancy outcomes: GrimAge acceleration is associated with shorter gestational length and lower birthweight. Clin. Epigenet. 12, 120. doi: 10.1186/s13148-020-00909-2 32762768 PMC7409637

[B10] Sajdel-Sulkowska E. M. (2023). The impact of maternal gut microbiota during pregnancy on fetal gut–brain axis development and life-long health outcomes. Microorganisms 11(9), 2199. doi: 10.3390/microorganisms11092199 37764043 PMC10538154

[B11] Schoorlemmer J. Macias-Redondo S. Strunk M. Ramos-Ruíz R. Calvo P. Benito R. . (2020). Altered DNA methylation in human placenta after (suspected) preterm labor. Epigenomics 12, 1769–1782. doi: 10.2217/epi-2019-0346 33107765

[B12] Sereti I. Verburgh M. L. Gifford J. Lo A. Boyd A. Verheij E. . (2023). Impaired gut microbiota-mediated short-chain fatty acid production precedes morbidity and mortality in people with HIV. Cell Rep. 42, 113336. doi: 10.1016/j.celrep.2023.113336 37918403 PMC10872975

[B13] Tang M. Weaver N. E. Frank D. N. Ir D. Robertson C. E. Kemp J. F. . (2022). Longitudinal reduction in diversity of maternal gut Microbiota during pregnancy is observed in multiple low-resource settings: Results from the Women First trial. Front. Microbiol. 13. doi: 10.3389/fmicb.2022.823757 35979501 PMC9376441

[B14] Wang G. Xu R. Zhang B. Hong X. Bartell T. R. Pearson C. . (2023). Impact of intrauterine exposure to maternal diabetes on preterm birth: fetal DNA methylation alteration is an important mediator. Clin. Epigenet. 15, 59. doi: 10.1186/s13148-023-01473-1 37029435 PMC10082529

[B15] World Medical Association (2013). World Medical Association Declaration of Helsinki: ethical principles for medical research involving human subjects. Jama 310, 2191–2194. doi: 10.1001/jama.2013.281053 24141714

[B16] Yu J. Peng T. Lu J. Li X. T. Hu R. (2021). Analysis of the microbiome based on 16S rRNA gene signature in women with preterm versus term birth. Reprod. Dev. Med. 5, 81–89. doi: 10.4103/2096-2924.320887 41788931

